# Sensory substitution reveals a manipulation bias

**DOI:** 10.1038/s41467-020-19686-w

**Published:** 2020-11-23

**Authors:** Anja T. Zai, Sophie Cavé-Lopez, Manon Rolland, Nicolas Giret, Richard H. R. Hahnloser

**Affiliations:** 1 Institute of Neuroinformatics, University of Zurich and ETH Zurich, 8057 Zurich, Switzerland; 2 Neuroscience Center Zurich (ZNZ), University of Zurich and ETH Zurich, Zurich, Switzerland; 3 Institut des Neurosciences Paris Saclay, CNRS, Université Paris Saclay, Orsay, France

**Keywords:** Auditory system, Operant learning, Motivation, Reward, Birdsong

## Abstract

Sensory substitution is a promising therapeutic approach for replacing a missing or diseased sensory organ by translating inaccessible information into another sensory modality. However, many substitution systems are not well accepted by subjects. To explore the effect of sensory substitution on voluntary action repertoires and their associated affective valence, we study deaf songbirds to which we provide visual feedback as a substitute of auditory feedback. Surprisingly, deaf birds respond appetitively to song-contingent binary visual stimuli. They skillfully adapt their songs to increase the rate of visual stimuli, showing that auditory feedback is not required for making targeted changes to vocal repertoires. We find that visually instructed song learning is basal-ganglia dependent. Because hearing birds respond aversively to the same visual stimuli, sensory substitution reveals a preference for actions that elicit sensory feedback over actions that do not, suggesting that substitution systems should be designed to exploit the drive to manipulate.

## Abstract (web summary)

The challenge of sensory substitution as a therapeutic approach is to design systems that are well accepted by subjects. Here, in deaf songbirds, the authors substitute hearing with vision, suggesting substitution devices could provide sensory feedback for the key actions that are deprived.

## Introduction

Sensory substitution is a method of transforming stimuli from one sensory modality into another one^1^. Such transformation can be used as a therapeutic approach towards restoring perception from a defective sensory modality^2^. This approach has gained much interest in recent years thanks to both advances in technology and the remarkable cross-modal flexibility of the central nervous system^3–5^. However, one of the main obstacles hindering the wide adoption of substitution devices has been the amount of training necessary to make use of the new sensory input; in fact, blind subjects often give up using a substitution device before reaching a reasonable proficiency level because they feel overwhelmed and frustrated^4^.

How can this situation be remedied, and which are the general design principles that need to be respected for sensory substitution to be willingly adopted? Currently, the motivational consequences inherent in sensory substitution are poorly understood, partly because we are lacking a theory that would predict how a subject will respond to substituting input. One key question is whether substitution will increase or decrease the affective valence of a given motor action^6,7^. Ideally, we would like to know beforehand about actions that will suffer from a decrease in valence and therefore will be avoided by subjects. Vice versa, if we could predict the actions that will experience a boost in valence from substitution, we could provide better treatments to support skilled behaviors such as speech in the deaf.

The key question seems to revolve around which of the motivational systems is best served by substitution? One idea is that sensorially deprived subjects desire highly informative feedback about their actions. For example, substituting input could help subjects to reduce uncertainties inherent in their motor output and allow them to make better action choices. Accordingly, the artificial sensory input should perfectly differentiate among distinct action outcomes. In other words, substitution may elicit the desire to explore^8–10^, which is to seek knowledge about actions’ effects. According to this knowledge-seeking view, subjects will preferentially choose actions with uncertain outcomes^11^ or high predicted information gain^12–14^.

Another idea is that adaptive responses to substitution may focus on the intrinsic goal of manipulating the environment^15^ rather than to obtain knowledge. A manipulation drive can manifest for example as playful behavior observed in diverse vertebrates across mammals, birds, and reptiles^16–19^. According to this drive, subjects may be drawn towards actions for the sole reason that the latter triggers a significant sensory input. Substitution could thus uncover a desire to achieve some form of impact^20^, which is to preferentially choose actions with a noticeable effect.

**Fig. 1:**
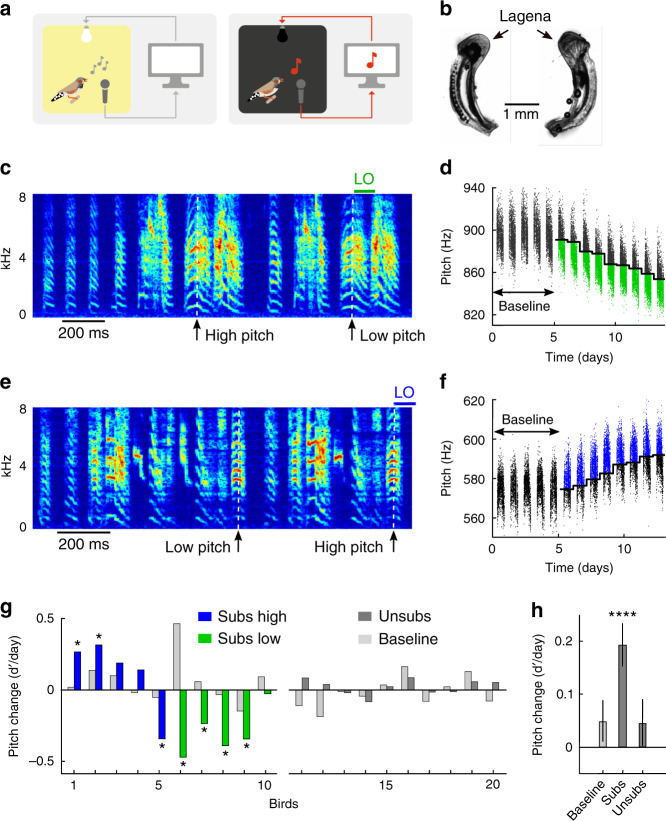
Light-off stimuli are positive reinforcers of vocal pitch in deaf songbirds. **a** Schematic of the experiment. A singing deaf bird inside a sound-isolation chamber (left) experiences a light-off (LO) stimulus for a duration in the range of 100–500 ms (right) when the pitch of one of its song syllables (red note) exceeds a given threshold (Credit: Sarah Steinbacher, MELS UZH). **b** Example picture of a pair of surgically removed cochleas. Complete deafness was confirmed by the presence of the osseous spiral lamina and by verification of an intact loop including the lagena. **c**, **e** Example song spectrograms in birds b2y2 (**c**) and b2p19 (**e**) with substituted feedback for low-pitched (**c**) and high-pitched (**e**) syllable renditions. The time points of pitch measurement are indicated by white dashed lines and the LO stimuli by green (**c**) and blue (**e**) bars. **d**, **f** Pitch values for syllable renditions without substitution (black dots) and with substitution (green dots: low-pitch subs, **d**); blue dots: high-pitch subs, (**f**)). The birds adapted the pitch in the direction of increasing LO rate. **g** Histograms of average daily pitch changes during substitution in birds with high-pitch substitution (subs high, blue, *n* = 5 birds, the first bar corresponds to b2p19 shown in (**e**) and (**f**)), low-pitch substitution (subs low, green, *n* = 5 birds, the 8^th^ bar corresponds to b2y2 shown in (**c**) and (**d**)), and in deaf control birds without substitution (unsubs, dark gray, *n* = 10 birds). The light gray bar to the left of each colored bar indicates the average daily pitch change in that bird during the last 5 baseline days. The asterisks indicate subs birds with significant pitch changes compared to controls (two-sample, two-sided *t*-test, *p* < 0.05). **h** Subs birds, as a population, adapt pitch in the direction of substituted feedback. Shown are the three fixed-effect terms of a mixed linear-effect model and their standard errors (282 observations from *n* = 10 subs and *n* = 10 unsubs birds). The bars indicate the daily change in pitch (d’/day) during baseline, during substitution in the direction of increasing light-off rate (subs, **** indicates nonzero fixed effect 0.19 d’/day, *p* = 3.0 × 10^−6^, SE = 0.04, tstat = 4.77, df = 279, confidence interval 0.11–0.27 d’/day, *n* = 20 birds), and in control (unsubs) birds.

To test whether knowledge-seeking or impact-seeking better explains adaptive responses to sensory substitution, in songbirds, we partially replace auditory feedback from a complex vocal behavior by visual feedback. We modified a widely applied operant conditioning paradigm involving the pitch of a song syllable. Instead of using short white-noise bursts played through a loudspeaker^21,22^, we substitute auditory feedback by visual feedback by briefly switching off the light in the sound-isolation chamber of the singing bird whenever the pitch of a targeted syllable was below (or above) a threshold (Fig. 1). We set the pitch threshold for light-off (LO) every morning to the median pitch value on the previous day. We investigated whether adult male zebra finches deafened using bilateral cochlea removal respond to such pitch substitution by LO. We evaluated birds’ responses to substitution in terms of d’ values, which are average daily pitch changes normalized by their standard deviations (see Methods). From these values, we inferred the affective valence of substituted feedback: whether it is neutral, aversive, or appetitive.

## Results

### Substituted feedback appetitively reinforces pitch

Because deafening by itself may induce a slow pitch drift with a nonzero bias^23,24^, we evaluated pitch responses to LO in comparison to responses in unsubstituted deaf control (unsubs) birds. Pitch changes in 7/10 subs birds significantly deviated from the drift in control animals in matched time periods (*p* < 0.05 in 7 of 10 subs birds, two-sample, two-tailed t-test of pitch change per day, see “Methods”, Fig. 1g).

Interestingly, subs birds tended to be attracted by LO, because of all birds except one changed pitch in the direction of increasing LO rate, Fig. 1g. If the direction of pitch drift were random in each bird with probability ½ in each direction (binomial model), then 9 of 10 birds would drift in the same direction in <1% of cases, corresponding to a *p*-value smaller than 0.01, suggesting that the pitch attraction by LO events was a non-random effect.

This simple preceding analysis, by inspecting only a binary value in each bird, is robust to details of the pitch measurement process. We obtained the same result when we fitted mixed linear-effect models to the pitch data, which can account for variability across individuals. The models contained three fixed terms: one term for the early time period before substitution (baseline) and one term each for the late time periods in subs and unsubs birds. In addition, there was one random term for each bird. We found that relative to baseline, subs birds exhibited pitch changes of 0.19 d′/day in the direction of increasing LO rate (nonzero fixed effect, *p* = 3.0 × 10^−6^, SE = 0.04, tstat = 4.77, df = 279, *n* = 20 birds, 100% of random pairings between subs and unsubs birds yielded *p* < 0.05, Fig. 1h), whereas unsubs birds did not change pitch (fixed effect 0.04 d′/day, not different from zero, *p* = 0.30, SE = 0.04, tstat = 1.04, df = 279, *n* = 20 birds, 0% of pairings yielded *p* < 0.05).

Syllables in deaf birds remained relatively stable over the short period of the experiment. Differential changes between sub-high and sub-low birds were specific to pitch but did not affect other sound features (*p* > 0.05, two-tailed *t*-test, duration, frequency modulation, amplitude modulation, and entropy, see “Methods”, Supplementary Fig. 1a). In combination, these results indicate that in deaf birds, substituted feedback is an appetitive reinforcer of song.

### The same LO stimulus tends to aversively reinforce pitch in hearing birds

**Fig. 2:**
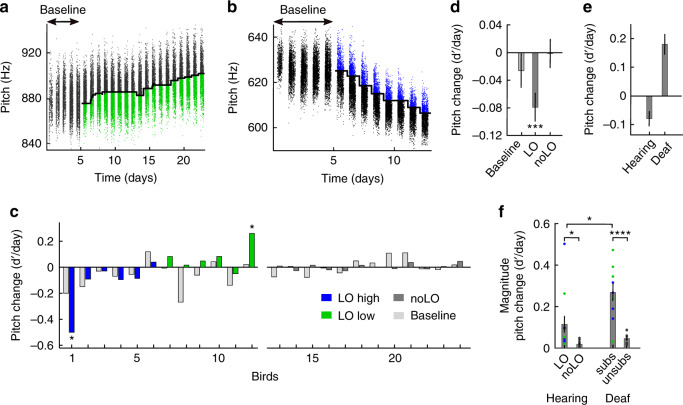
In hearing birds, the valence of light-off reinforcers is negative. **a**, **b** Hearing birds change pitch in the direction of decreasing LO rate, here shown for a low-pitch light-off (LO low) bird (bird b2y2, **a** same bird as in Fig. 1c, d) and a LO high bird (bird p6s6, **b**). Legend as in Fig. 1d, f. **c** Histograms of average daily pitch changes in LO high (blue, *n* = 6), LO low (green, *n* = 6), and in hearing control (noLO) birds (gray, *n* = 12). The asterisks indicate subs birds with significant pitch changes compared to controls (two-sample, two-sided *t*-test, *p* < 0.05, see “Methods”). **d** The three mixed linear fixed effect terms and their standard errors. The bars indicate the daily change in pitch (d’/day) during baseline, during LO exposure in the direction of increasing LO rate (LO, ***indicates non-zero fixed effect −0.08, *p* = 1.4 × 10^−4^, df = 377, SE = 0.02, tstat = −3.85, confidence interval −0.12 to −0.04 d’/day, *n* = 24 birds), and in hearing control birds (noLO). **e** Average directed pitch changes over all days in LO (hearing, 144 days) vs subs (deaf, 102 days) birds. Hearing birds changed their pitch away from the LO zone (decreasing the number of renditions with LO) and deaf birds towards the LO zone (increasing the number of renditions with LO). The error bars indicate the standard errors of the mean. **f** The magnitude of average pitch change is larger in subs birds than in LO birds (0.16 d’/day, *indicates *p* = 0.01 for average magnitude, tstat = 2.73, df = 20, two-sample two-sided *t*-test, *n* = 12 LO and *n* = 10 subs birds), and much larger than in unsubs birds (0.22d’/day in time-matched periods, **** indicates *p* = 4 × 10^−5^, tstat = −5.35, df = 18, *n* = 20 birds, two-sample two-tailed *t*-test). The magnitude of average pitch change is larger by 0.10 d’/day for hearing LO than noLO birds (*p* = 0.02, tstat = −2.43, df = 22, two-sample two-tailed *t*-test, *p*-values are not adjusted for multiple comparisons. The error bars indicate standard errors of the mean and the dots represent individual birds (colors as in c).

We also evaluated adaptive pitch responses in hearing birds. A small number of hearing birds responded to LO: Pitch changes in two of 12 birds significantly exceeded the spontaneous pitch drift in hearing controls (noLO, *p* < 0.05, two-tailed *t*-test on pitch changes per day, see “Methods”, Fig. 2a–c). A mixed linear effect model revealed that hearing birds significantly changed pitch in the direction of decreasing LO rate (−0.08 d′/day in the direction of LO, nonzero fixed effect of LO, *p* = 1.4 × 10^−4^, df = 377, SE = 0.02, tstat = −3.85, *n* = 24 birds including 12 controls, Fig. 2d, 100% of random pairings between LO and noLO birds revealed a significant non-zero fixed effect of LO), implying that overall, LO was aversive in hearing birds (the fixed effect for baseline was not significantly different from zero, *p* = 0.33, and neither was the fixed effect for noLO, *p* = 0.95). In combination, our findings show that deafening causes an inversion of affective valence of LO reinforcers, Fig. 2e.

To analyze the sensitivity of birds’ vocal response irrespective of whether they were attracted or repelled by LO, we quantified their magnitude pitch responses as the normalized pitch change per day (d′ value) aligned in the direction of global pitch change, implying that the average magnitude change was always a positive number. The daily magnitude pitch change was larger by 136% in deaf birds compared to hearing birds (difference 0.16 d′/day, *p* = 0.01, tstat = 2.73, df = 20, *n* = 12 hearing and *n* = 10 deaf birds, two-tailed *t*-test, Fig. 2f). Thus, visual feedback is much more salient when it substitutes auditory feedback in deaf animals than when it is a supplemental feedback in hearing birds.

**Fig. 3:**
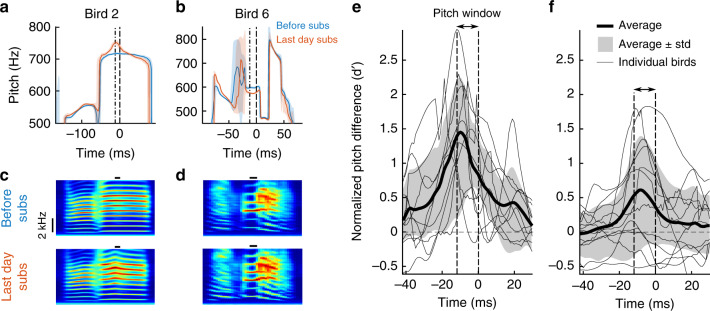
Within-syllable pitch trajectories show time-localized learning. **a**, **b** median (solid line) and quantiles (shaded area) of within-syllable pitch during baseline (blue) and on the last day of substitution (last day subs, red) in a subs-high (**a**) and a subs-low bird (**b**). The two dashed vertical lines show the window within which we calculated pitch to determine whether to switch off the light or not. The bird numbers correspond to target syllable numbers in Fig. 1g. **c**, **d** Average spectrogram of the targeted syllable before (top row) and after (bottom row) the substitution paradigm. Same time axis as in (**a**). The horizontal black bar indicates the pitch calculation window. **e**, **f** Traces of normalized pitch differences between (the last day of) baseline and the last day of subs in deaf subs (**e**) and in hearing LO birds (**f**). Birds adapted pitch within about 10 ms of the pitch calculation time window (delimited by dashed vertical lines). The fine curves show pitch difference traces in individual birds, the thick curve their average, and the gray area indicates the average ± one standard deviation. Curves in birds that decreased pitch are flipped to make pitch changes positive.

Although they responded to LO in opposite directions, deaf and hearing birds similarly modified their songs only in a very narrow time window, their maximum adaptive pitch changes were mainly confined to within roughly 10 ms of the targeted time window for LO delivery, Fig. 3 and Supplementary Fig. 2.

### Effects of substitution on singing rate

**Fig. 4:**
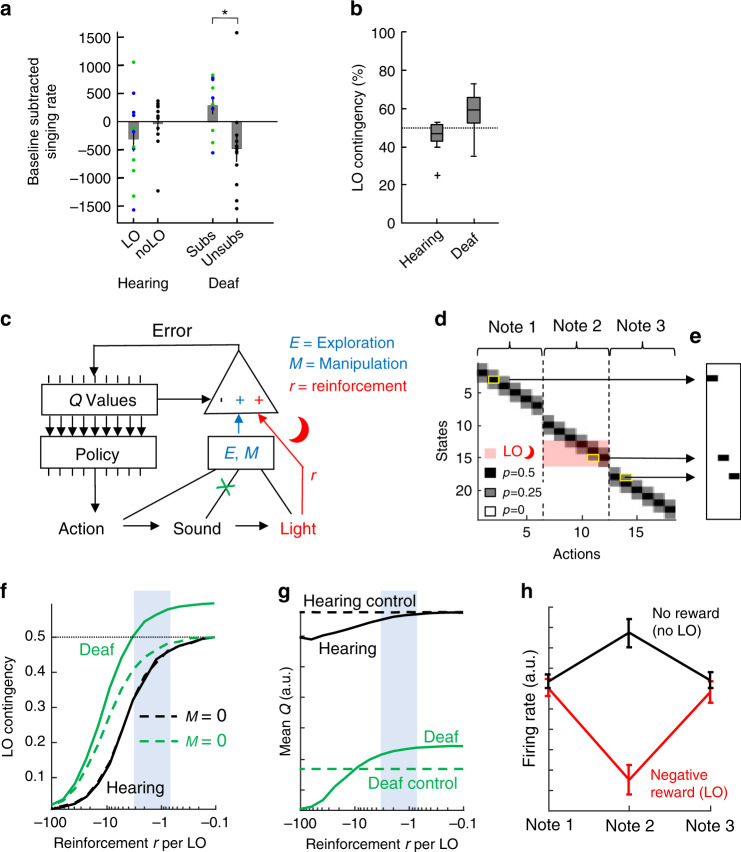
A manipulation bonus is compatible with valence inversion. **a** Average change in singing rate during the last three days of light off in deaf (*n* = 10) and in hearing birds (*n* = 12) and in their time-matched controls (*n* = 12 deaf and *n* = 12 hearing birds). The change is reported relative to the average on the last three days of baseline (* indicates *p* = 0.02, tstat = −2.65, df = 20, two-sample two-tailed *t*-test). The error bars indicate standard errors of the mean and the dots indicate individual birds (blue/green for light off high/low and black for birds that were not subjected to light off events). **b** Hearing (*n* = 12, LO) birds trigger light off on average in <50% of cases whereas deaf (*n* = 10, subs) birds do so on average in more than 50%. The center line of the boxplot represents the median, the box bound extends to the 25^th^ and 75^th^ percentile and the whiskers extend to the minima and maxima excluding outliers indicated as a black cross (outlier are defined according to MATLAB’s default definition as values that are more than 1.5 times the interquartile range away from the top or bottom of the box). **c** We modeled a simple agent that maximizes total reward formed by the sum of the extrinsic reinforcement *r* (red), an exploration bonus *E*, and a manipulation bonus *M* given by impact. The agent’s greedy policy is to choose the action with maximal *Q* value (expected total reward). Deaf birds receive no auditory input (green cross). **d** Markov model of an agent that generates one syllable composed of three consecutive notes, each associated with six possible variants (actions). An action triggers one of three possible sensory states with probabilities depicted with gray shading. States 13–16 trigger light-off (r**e**d). **e** Example syllable generated by the model (the underlying action-state pairs are delimited in yellow in **d**). **f** Hearing birds trigger light-off on <50% of syllables for all choices of negative reinforcement *r* per LO. Deaf birds reach above the critical level of 50% LO contingency (green), which is not the case when the manipulation bonus is zero (M = 0, dashed line). **g** Simulated subs birds are more motivated to sing than their controls (unsubs), their mean *Q* value (green, arbitrary units) is above that of unsubs birds (dashed green). In hearing birds, the situation is reversed, they are less motivated than their controls. The blue dashed area indicates the plausible reinforcement-per-LO region that qualitatively matches our results. **h** Model neurons’ firing rates (in hearing birds) agree with reward prediction error coding seen in dopaminergic neurons. On aversively reinforced trials during Note 2 (modeling a LO event or an acoustic white-noise stimulus), the firing rate decreases (red), whereas on escape trials (no reinforcer, no LO), the firing rate increases (black). Error bars depict mean ± standard deviations (across simulated model birds).

Valence inversion was also signaled by the contrasting effects of LO on singing rates (Fig. 4a). On the last three days of substitution, subs birds produced on average 291 more song motifs (average increase of 25%) than on the last three days of baseline, which deviated from their deaf controls (unsubs) that sung on average 479 fewer song motifs (average decrease of 34%, *n* = 10 subs and *n* = 12 unsubs birds, *p* = 0.02, tstat = −2.65, df = 20, two sample two-tailed *t*-test, 97% of random matchings resulted in a significant *p*-value). By contrast, hearing birds were oppositely but less affected by light off: LO birds produced 310 fewer song motifs on the last three days of LO than during baseline (average decrease of 9%), whereas their hearing controls produced 38 fewer song motifs (average increase of 8%, *p* = 0.29, tstat = 1.09, df = 22, *n* = 12 LO and *n* = 12 noLO birds, two sample two-tailed *t*-test, 0% of random pairs resulted in a significant *p*-value). As expected, when accounting for individual-level differences, there was a significant difference between the effect of LO on hearing and on deaf birds (non-zero fixed interaction between deafening and light off, *p* = 0.005, df = 134, tstat = 2.86 for assigned matches, *n* = 46 birds, 99.9% of random matching resulted in a significant interaction term), suggesting that substitution differentially affects deaf and hearing birds in their motivation to sing.

The increased motivation to sing caused by substitution should depend sensitively on the manipulability of LO. To explore whether the effect of light off on singing rate is due to our enforced link with performance, we conducted experiments in two deaf birds in which we delivered LO at a precise time in the song but irrespective of pitch performance (subs-rand birds). That is, we turned off the light in randomly chosen 50% of targeted syllables, regardless of pitch. After 11 days of LO exposure (corresponding to the average duration of LO exposure in subs birds, see “Methods”), the two subs-rand birds strongly reduced their singing rate to roughly 50% of the baseline rate (40 and 55%, Supplementary Fig. 3d), indicating that substitution is motivating when controllable. Deaf birds seem to prefer LO feedback that is predictable over unpredictable feedback.

### LO contingencies are aligned with pitch responses

Deaf and hearing birds exhibited different LO contingencies. While in hearing birds on average 46% of syllable renditions triggered LO (*p* = 0.07, tstat = −2.03, df = 11, two-tailed *t*-test of the hypothesis that LO rate is 50%), in deaf birds the average LO rate was 57% (*p* = 0.03, tstat = 2.63, df = 9, two-tailed *t*-test of the hypothesis that LO rate is 50%, Fig. 4b). Thus, deaf birds increased the LO contingency of their actions away from the 50% expectation set by the previous day whereas hearing birds decreased the LO contingency, which is aligned with the pitch responses in both bird groups.

### Valence inversion does not reflect a preference for darkness in deaf birds

A simple explanation of our findings could be that deafness induces an attraction to darkness for whatever reason. This explanation was ruled out after we replaced LO by light-on stimuli and found strongly appetitive responses to such stimuli in deaf birds (0.71 ± 0.07 d′/day, Supplementary Fig. 3a, b) in the direction of increasing light-on rate (one subs-high bird: 0.61 d′/day, one subs-low bird: 0.77 d′/day), 2/2 birds significantly exceeded spontaneous pitch drift in control (unsubs) birds, *p* < 0.05, two-tailed t-test on daily pitch changes, light-on contingency 77% ± 0.8% (75 and 78%), see “Methods”. We speculate that the affective valence of light-on seems to be so much larger than that of LO because the latter stimulus briefly disturbs birds in their locomotion planning whereas light-on seems more neutral in its intrinsic valence.

### A manipulation bonus can explain valence reversal

Our vocal-light substitution paradigm forms a simple but powerful touchstone for theories of intrinsic motivation because (1) the vocal space we imposed on deaf birds is essentially binary (light on vs off), (2) the environment has no intrinsic dynamics (light only depends on pitch), (3) there has been no evolutionary adaptation of pitch to LO stimuli, and (4) birds have no physiological need to sing a particular pitch (unlike their need of food intake for example). Despite this simple framework, most models of behavioral learning cannot accommodate valence inversion. In reinforcement learning (RL)^25^, stimuli have either appetitive or aversive effects and standard RL models cannot accommodate valence inversion for example via changes in baseline reward due to deafening^26,27^.

Our findings are also incongruent with computational models of directed exploration that involve an exploration bonus for action policies that are either informative^12–14^, diverse^8,9^, or simple^28^ (Table 1). These theories have been designed to either improve the efficiency of RL models or to model human behavior within a restricted class of multi-armed bandit problems^10,12,13^. In these models, agents choose actions that maximize the information gained about the environment, which is often modeled as an exploration bonus in proportion to the uncertainty of an action’s value^9,10^. Yet, in binary (and pitch-symmetric) worlds as ours, knowledge gain is maximal when agents uniformly sample from their action repertoire, implying that such theories predict the convergence of LO contingency to 50%^12,13,29^, which contradicts the divergence we found in subs birds.

**Table 1:** Reinforcement learning characteristics and their compatibility with valence inversion.

Reinforcement learning model characteristic	Additional info	Can it explain valence inversion?
Changes in baseline reward	Caused by deafening	No
Exploration bonus	For informative actions	No
	For diverse actions	No
	For simple actions	No
Manipulation bonus	For impact	Yes

We found that, to step above a LO contingency of 50%, a manipulation bias is required towards actions that impact the environment (such as light off). We introduced such a bias by defining a manipulation bonus *M*_*j*_ associated with action *j*. This bonus $M_j = D_{{\mathrm{KL}}}\left( {\hat \vartheta _0||\hat \vartheta _j} \right)$ models the impact of action *j* in terms of the Kullback-Leibler divergence between the estimated sensory probability density $\hat \vartheta _j$ following action *j* and the same density $\hat \vartheta _0$ without any preceding action. Let us denote the LO probability following action *j *by $\hat \vartheta _j({\mathrm{off}})$ and the LO probability without acting by $\hat \vartheta _0\left( {{\mathrm{off}}} \right)$. Because we imposed $\hat \vartheta _0\left( {{\mathrm{off}}} \right) = 0$, it follows that in deaf birds, the impact of action *j* is given by the Shannon surprise of light on: $M_j = - \log \hat \vartheta _j\left( {{\mathrm{on}}} \right)$. This impact is the larger the more likely it is that action *j* triggers LO (the smaller $\hat \vartheta _j\left( {{\mathrm{on}}} \right)$). By experimental design, the impact is nonzero only for a small set of LO-triggering actions. An agent that maximizes impact will therefore exhibit a (manipulation) bias towards LO. In hearing birds, by contrast, the sensory environment includes vision and audition. Thanks to auditory feedback, all vocalizations in hearing birds elicit a nonzero impact. Thus, when hearing birds maximize impact, no particular action is singled out, which leads to the absence of a manipulation bias towards light off in LO birds.

In simulations, we modeled birds’ intrinsic reward *R*_*j*_ = *E*_*j*_ + *M*_*j*_ + *r*_*j*_ associated with action *j* as the sum of an exploration bonus *E*_*j*_ (given by information gain), a manipulation bonus *M*_*j*_, and an extrinsic punishment *r*_*j*_ ≤ 0 associated with LO (*r*_*j*_ < 0 only in case of light off), Fig. 4c, d. We simulated a simple agent that maximizes *R*_*j*_ using SARSA^30,31^, a standard RL framework (see Supplementary Methods). We found that when the punishment *r*_*j*_ per LO was such that deaf birds’ LO contingency converged to values above and hearing birds’ to values below 50%, Fig. 4f, the singing preference increased in deaf birds and it decreased in hearing birds, compared to their simulated controls, Fig. 4g, in qualitative agreement with data. A manipulation bonus was required to reproduce these findings, Fig. 4f. Thus, when a behavioral goal is to detect impact via sensory feedback, such intrinsic reward can account for valence inversion and for high salience of substituted feedback. Furthermore, by design^32^, the model output in Fig. 4 agreed with known reinforcement-related firing behavior of dopaminergic neurons^33^, which in hearing birds fire less than average on escape trials (no negative reinforcement) and more than average on hit trials (negative reinforcement), Fig. 4h. This simple model also captures the behavior of subs-rand birds in that their maximal achievable impact is log 2 (because of unpredictability of LO events), which is lower than the impact that subs birds can achieve.

### Basal ganglia lesions prevent learning from substituted feedback

**Fig. 5:**
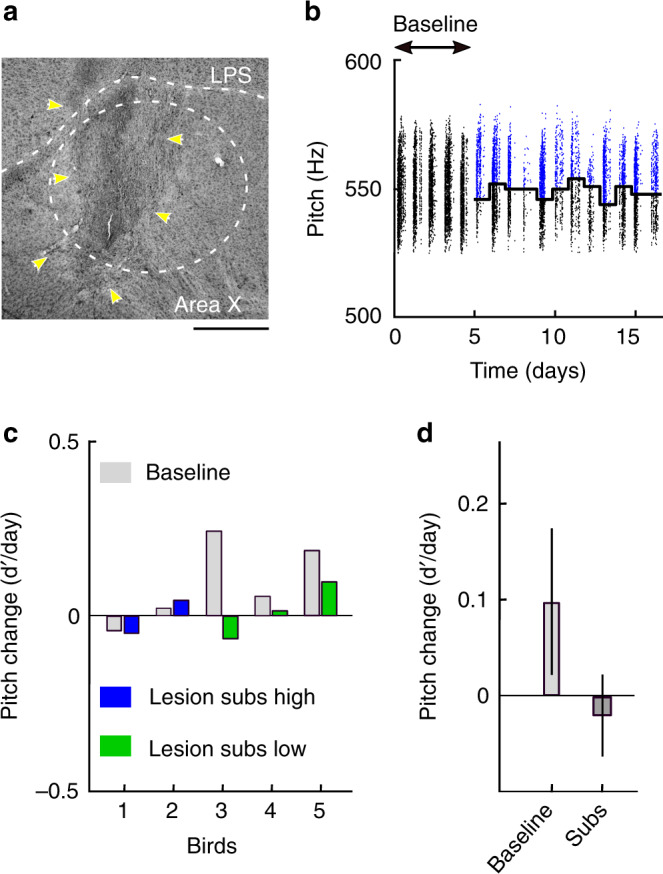
A basal ganglia pathway is necessary for adaptive responses to substituted feedback. **a** Example sagittal brain section of a bird with lesion (yellow arrows) in Area X (dashed white ellipse). The lamina pallio-subpallialis (LPS) is indicated by the white dashed line. The scale bar on the bottom right indicates 500 µm. Anterior is towards the left, posterior to the right. **b** Pitch values of all syllable renditions in an example deaf bird with bilateral lesions in Area X. There is no clear adaptive response to substitution. **c** The average pitch changes (d’/day) in deaf birds with Area X lesions during baseline (light gray), during high-pitch substitution (blue), and during low-pitch substitution (green). **d** The two fixed-effect terms of a mixed linear effect model and their standard errors: the daily pitch changes (1) during baseline (left bar, *p* = 0.21, tstat = 1.27, df = 83, *n* = 5 birds), and (2) during substitution in the direction of increasing LO rate (right bar, *p* = 0.64, SE = 0.04, tstat = −0.47, df = 83, *n* = 5 birds) are not significantly different from zero (not indicated in the figure).

Our RL model suggests an involvement of the basal ganglia in mediating a manipulation bias. Dopaminergic neurons can drive selective pitch changes via their action in Area X^34–36^, a region homologous to the mammalian basal ganglia^22,37^. To probe for a manipulation bias in Area X, we made irreversible bilateral lesions in Area X of deaf birds. When these birds were subjected to substituted LO feedback, none of them (*n* = 5) changed pitch in excess to deaf controls (*p* > 0.05 for all birds, two-sampled *t*-test) (Fig. 5a–c). One bird in which the lesion did not overlap with either Area X or LMAN in both hemispheres changed pitch significantly compared to deaf controls (*p* = 0.01, two-sampled t-test). In lesioned subs birds, the magnitude of average pitch change per day was smaller than in unlesioned subs birds (difference −0.22 d′/day, *p* = 0.003, tstat = −3.62, df = 13, two-tailed two-sample *t*-test), and the daily pitch change in lesioned subs birds was not significantly different from zero (−0.02 d′/day, *p* = 0.64, SE = 0.04, tstat = −0.47, df = 83, *n* = 5 birds, fixed effect) (Fig. 5d). Similarly to subs birds, lesioned subs birds have a tendency to produce on average more song motifs on the last three days of substitution (154 song motifs or 41% more) than on the last three days of baseline, Supplementary Fig. 3d. In combination, these findings show that Area X is necessary for expressing adaptive pitch responses to substituted feedback.

## Discussion

Our finding that the song system is able to assign a cross-modal light stimulus the role of an instructive pitch-biasing motor signal helps to refine our understanding of the neural basis of vocal learning. Namely, because targeted changes of vocal skills can occur without hearing, it follows that evaluation of auditory performance is not a prerequisite for vocal plasticity in adulthood, unlike commonly assumed^33,37^. Our findings do not rule out the use of vocal performance for template-based song learning^38^, but they showcase that some forms of vocal learning do not rely on auditory representations of song, and that pathways not concerned with audition are able to efficiently operate on the brain’s motor representations of song. The high efficiency and temporal precision of light-instructed pitch changes agree with previous observations that binary feedback signals can promote robust motor learning^39,40^.

To enable flexible assignment of visual signals (light intensity) to specific motor features (pitch), the visual system must feed into the song system in a computationally powerful way. Not much is known about the neural circuits that provide substituting visual stimuli to motor centers, but we find that the cross-modal learning circuit involves the basal ganglia, which provides some clues as to the neural mechanisms underlying substituted motor learning. For one, given that cerebral neurons efferent to the basal ganglia do not even respond to auditory feedback during singing^41–43^, it is unlikely that multimodal visual-vocal neurons are involved in cross-modal learning. Rather, a large body of work on the basal ganglia evidences an error-like signal that reinforces time-resolved motor representations of song^33,35,44^. Our work therefore suggests that the avian basal-ganglia part of the song system has evolved to support multimodal learning independent of the sensory modality of reinforcement.

Our key finding is that elimination of auditory feedback induces appetitiveness of an otherwise aversive visual reinforcer of song. This finding is unrelated to whether deaf birds perceive light stimuli differently than hearing birds, as we did not probe behavioral responses to visual input other than via song. Rather, the missing feedback seems to unleash the need for a substitute, uncovering a strong drive to manipulate sensory input. Such a manipulation drive better explains our observations than intrinsic motivations such as activity and exploration^15^. Our interpretation is that normally, in healthy vocalizers, the need to manipulate is satisfied and does not constrain the brain’s valence system. However, when sensory feedback is lacking, this need becomes overwhelming to the point that it can override the valence even of aversive stimuli. In our view, this remarkable dictatorship of the manipulation drive emphasizes one of the most basic needs associated with motor actions, which is to perceive sensory feedback.

Perhaps these insights provide us with new low-level clues about the function of song. By design, the slightly aversive LO events are the only feedback signals that deaf birds experience, which is why to satisfy their manipulation drive, they prefer it over no response at all (something is preferable for a curious agent over nothing). One function of song in birds may thus be to exert an influence on the environment to signal the singer’s presence, even if confirmed only by visual feedback as in our experiment. Birds’ tendency to avoid overlaps in their vocalizations^45^ is independent evidence for their determination to maximize the control (impact) over the acoustic space during a vocalization.

In humans, there exists a compelling analogy of this remarkable alteration of affective stimulus valence. Namely, a manipulation drive shows up during boredom, which can prompt subjects to display behaviors that evidence paradoxical preference of otherwise aversive stimuli^46,47^. Lacking an alternative, subjects prefer an unpleasant experience rather than none at all.

Our findings strengthen the view that the frustration experienced by users of many substitution devices could be linked with the level of uncontrollability of the substituting input. By generalization, users might avoid motor actions when these do not elicit some form of substituting sensory feedback. Although subjects could be initially drawn towards information seeking when using a new device, once this drive saturates (which is assumed to happen early on for simple binary feedback), action selection will become dominated by the manipulation drive. Substitution system should therefore not be designed to maximize information, especially when information maximization interferes with a manipulation bias that draws the use of a device away from its ill-defined purpose.

By contraposition, to meet the needs of sensorily deprived subjects, substitution devices should provide feedback about motor actions and they should let subjects feel empowered through the new sense. One promising approach is to design substitution systems as part of closed sensorimotor loops^4^, and ideally these systems would stimulate motor learning, which can be fun as in tennis practice or piano playing rather than strenuous as in learning a foreign language (analogies between learning to make use of substituted input and reading have been drawn^1^). Perhaps, acceptance of substitution devices would also increase when training setups are designed to let subjects predictably manipulate the substituting sensory input, in line with insights from interviews conducted with users of substitution devices^48^.

Exploiting the manipulation drive in substitution therapies need not negatively impact information gain; rather, a manipulation bias may be beneficial when this bias points towards desirable actions. For example, the blind might benefit from a signal that reports the inverse distance of the hand to an object of choice. Or, in the context of speech rehabilitation, the hearing impaired may benefit from short-latency feedback when their variable speech agrees with signals of high comprehensibility; such feedback could be provided as visual signal (e.g., displayed in augmented reality devices) or as vibro-tactile signal^49^. One requirement for such an idea to be effective is that the manipulation drive that we observed for single actions will generalize to action sequences such as multi-syllabic patterns.

Manipulation biases might also be relevant in neuroprosthetic systems that aim to increase the perceptual space of subjects. For example, in sensory neuroprosthetics, the sensor is not substituted but bypassed by electrical stimulation of downstream neurons. While neuroprosthetic closed-loop systems have only recently started to be explored in the sensory domain^50^, closed-loop systems are very common in the motor domain^51^, where animal models have played a crucial role in the development of a wide range of those systems^51,52^. Closed-loop motor systems achieve better performance than open-loop systems^51,52^ and there is a distinct performance benefit of high feedback rates^53^. These facts lend support to the idea that sensory neuroprosthetic systems will also benefit from closed-loop design. In this regard, the zebra finch may lend itself as an ideal animal model for exploring closed-loop approaches to sensory neuroprosthetics^54^.

We believe that when the manipulation drive is abstracted as an action-selection principle of a software agent, such a drive can serve key computational functions. Namely, under some circumstances, manipulation seeking can be preferable to knowledge seeking because the latter is uninformative about relevance. For example, a manipulation drive can prevent an agent from getting stuck in front of a computer screen displaying random stimuli, which would otherwise be the most absorbing stimulus for a purely knowledge-driven agent that does not distinguish between self-generated and external stimuli. It is therefore not surprising that concepts such as manipulation and impact are gaining in importance in machine learning. In a recent curiosity-driven RL approach, it was found that a focus of actions on self-generated sensory feedback can dramatically expedite learning progress^55^.

Further impulses for understanding the motivational drives behind spontaneous behaviors are strongly needed. Although we modeled the manipulation drive as the simple desire to maximize the distance between the world models with and without acting, other formulations of manipulation with similar effect are imaginable, for example based on empowerment^29,56^.

We propose that sensory substitution is a promising paradigm not just to experimentally characterize the motivation to manipulate, but also to dissect the neural representations of affective valence^57^ and to probe how substituting input is integrated into an existing circuit on the level of single cells, which so far is only understood on the level of brain areas^1,58^. Because the manipulation drive seems to have access to cross-modal learning mechanisms that are as fast and efficient as those of normal motor learning, sensory substitution and the manipulation drive it reveals may provide further glimpses on some of the enabling factors of successful evolutionary adaptations.

## Methods

### Subjects and song recordings

We used 55 adult male zebra finches (*Taeniopygia guttata*) raised in our breeding facilities in Zürich (Switzerland) and Orsay (France). At the beginning of the experiment, birds were between 90 and 200 days old. During the experiment, birds were housed individually in sound-attenuating recording chambers on a 14/10-h day/night cycle. Access to food and water was provided *ad libitum*. After 2–5 days of familiarization in the experimental environment, birds resumed singing at a normal rate. Songs were recorded with a wall-attached microphone, band-pass filtered, and digitized at a sampling rate of 32 kHz. All experimental procedures were approved by the Veterinary Office of the Canton of Zurich and by the French Ministry of Research and the ethical committee “Paris-Sud et Centre” (CEEA No. 59, project 2017-12).

### Visual substitution of pitch

To provide pitch substitution, we ran a custom LabVIEW (National Instruments, Inc.) program. We targeted a harmonic syllable using a two-layer neural network trained on a subset of manually clustered vocalizations. We evaluated pitch (fundamental frequency) in a 16-ms window at a fixed delay after the syllable detection point (which occurred at a roughly constant time lag after syllable onset). We estimated pitch using the Harmonic Product Spectrum algorithm^59,60^.

Following pitch estimation, we provided pitch substitution by switching off the light (using a relay) in the sound recording chamber after a delay of 12 ms and for a duration in the range between 100 and 500 ms. Two birds were held in dim light and instead of switching off the light, we provided substitution by turning on an additional light. We substituted either high or low pitch depending on a manually set threshold. In two birds we randomly delivered substitution in 50% of detected syllables, independently of the pitch measurement.

To cumulatively drive the pitch of the targeted syllable away from the baseline, every morning, we adjusted the pitch threshold to the median pitch value on the previous day, where we computed the median on all noncurated neural network detections (in 24/328 days from 15/29 birds, we did not set the threshold to the median value because of a software crash on the previous day). In 15 birds (six hearing and nine deaf, among which three received brain lesions), we delivered substitution on high-pitched syllable renditions, and in 15 birds (six hearing and nine deaf, among which three received brain lesions) we delivered substitution on low-pitched syllable renditions. Subs birds were deaf birds exposed to LO substitution, unsubs birds were deaf and unsubstituted birds; LO birds were hearing and exposed to LO feedback, and noLO birds were hearing and not exposed to LO; subs-rand were deaf birds exposed to random LO events.

### Surgeries

Before the onset of surgery, we provided analgesia with the nonsteroidal anti-inflammatory drug carprofen (2–4 mg/kg, Norocarp, ufamed AG, Sursee, Switzerland) given intra muscularly (IM). Birds were deeply anesthetized using isoflurane (1.5–3%) and placed in a stereotaxic apparatus. We applied the antiseptic povidone-iodine (Betadine, Mundipharma Medical Company, Basel, Switzerland) to the skin at the incision site, followed by the local anaesthetic lidocaine in gel form (5%, EMLA, AstraZeneca AG, Zug, Switzerland).

### Deafening procedure

In the stereotax, the head angle formed by the flat part of the skull above the beak and the table was set to 90°. The skin was opened above the hyoid bone and the neck muscles were gently pushed back to expose the semi-circular canals. A hole was made in the skull to access the inner ear below the semi-circular canals. The cochlea was visually identified based on the surrounding bone structure and a small hole was made with forceps into the cochlear base. We removed the cochlea from the cavity with a custom-made tungsten hook and took a picture of both intact cochleas including the lagenas to document the success of the procedure (Fig. 1b).

### Area X lesions

We set the head angle formed by the flat part of the skull above the beak and the table to 35° and drilled a window into the skull above Area X. Area X was localized based on stereotaxic coordinates and identified through the presence of tonically firing neurons, recorded with a 0.6–1.7 MΩ tungsten electrode attached to a vertical manipulator. In each hemisphere we injected 1 μl of ibotenic acid (Tocris) near the center of Area X. Injection sites were located on average 1.5–1.9 mm medial-lateral (ML), 5.5–6.0  mm anterior-posterior (AP), and 2.8–3.5 mm dorsal-ventral (DV) from the bifurcation of the midsagittal sinus (lambda). Injections were performed using a borosilicate glass pipette (BF-120-69-10, Sutter instrument) pulled with a Picospritzer (Parker Inc.) and broken with forceps to a tip diameter of about 10 μm.

### Histology

At the end of the experiment, birds were euthanized with an overdose of intraperitoneal injection of sodium pentobarbital (200 mg/kg, Esconarkon, Streuli Pharma AG, Uznach, Switzerland) and intracardially perfused with 4% paraformaldehyde (PFA) before brains were removed for histological examination. Brains were rinsed in a 0.01 M phosphate buffer solution. The hemispheres were separated from each other, glued on a metal plate, and embedded in 3% agar. Sagittal slices of 80-μm thickness were cut with a Thermo Microm HM650V microtome and mounted on slides for Nissl staining.

### Statistical pitch analysis

We curated the neural network detections manually by visually removing misdetections (triggered by noises or similar vocal patterns not corresponding to the targeted syllable). We quantified the effects of LO on the pitch using d-prime values $d^{\prime}_{i,j}$:

$$d^{\prime}_{i,j} = \frac{{\bar p_j - \bar p_i}}{{\sqrt {\frac{1}{2}\left( {\sigma _i^2 + \sigma _j^2} \right)} }},$$

where $\bar p_i$ and $\bar p_j$ are the mean pitches of the curated syllable on days *i* and *j*, and $\sigma _i^2$ and $\sigma _j^2$ are the respective pitch variances.

### LO start criterion

LO started after at least 5 days of stable singing ($\left| {d^{\prime}_{i - 4,i}} \right| < 0.5$ with *i* being the last day of baseline); in two birds, LO started earlier and in one bird, LO started later because of technical issues and unforeseen scheduling constraints. In deaf (subs) birds, LO started on average 16 days after deafening (range 9 days for bird 6 in Fig. 1g to 34 days for bird 3 in Fig. 1g) and in hearing (LO) birds it started after at least 7 days in isolation.

In deaf birds, we did not find a significant correlation between the number of days between deafening and substitution onset and the absolute average pitch change per day (*R* = −0.21, *P* = 0.57) nor between the number of days since deafening and the maximum pitch change away from baseline during substitution (*R* = −0.09, *P* = 0.81).

### LO end criterion

We ended the LO paradigm (in both deaf and hearing birds) when the absolute mean pitch change (relative to baseline) either exceeded 2.5* d*′ or when it stabilized near zero, which was defined as $\left| {d^{\prime}_{i - 4,i}} \right| < 0.5$ with the index *i* referring to the last day of light off (in one bird, we ended substitution before this criterion was met because the song degraded too much for reliable syllable detection). The duration of the LO paradigm did not differ significantly between hearing and deaf birds (*p* = 0.37, two-tailed two-sample *t*-test, mean hearing = 13 days, mean deaf = 11 days). Thus, the observed differences between hearing and deaf birds in Fig. 2e, f were not due to differences in time spent in the experimental chamber.

The average daily pitch change $\overline {d^{\prime}_{{\mathrm{LO}}}}$ during substitution in each animal (Figs. 1g and 2c) we quantified as $\overline {d^{\prime}_{{\mathrm{LO}}}} = \left\langle {d^{\prime}_{i - 1,i}} \right\rangle _i$, where the angle brackets denote averaging across all days *i* with LO (starting from the second day).

Similarly, the average daily pitch change $\overline {d^{\prime}_{\mathrm{B}}}$ during the baseline period in each animal (light gray bars in Figs. 1g and 2c) we quantified as $\overline {d^{\prime}_{\mathrm{B}}} = \left\langle {d^{\prime}_{i - 1,i}} \right\rangle _i$, where the average runs across the last 4 days *i* before LO.

### Magnitude pitch change

We assessed the magnitude pitch change in each bird irrespective of its preference (attraction or repulsion by LO). To discount for preference, we first defined the global direction *δ* of pitch change during LO as $\delta = {\mathrm{sign}}\left( {d^{\prime}_{b,l}} \right)$, where *b* is the last day of baseline and *l* is the last day of LO exposure (*δ* corresponds to the direction of the colored bars in Figs. 1g and 2c). Thus, if birds shifted pitch upward towards higher values, *δ* = 1, and if birds shifted pitch down, *δ* = −1. In each animal, we computed the mean aligned pitch change $\overline {a^{\prime}}$ during substitution as the average daily change $d^{\prime}_{i - 1,i}$ multiplied with *δ*: $\overline {a^{\prime}} = \delta \cdot \left\langle {d^{\prime}_{i - 1,i}} \right\rangle _i$, (*i* = 6,..., end). Figure 2f shows $\overline {a^{\prime}}$ averaged over all birds. For control birds (unsubs, noLO), the direction of change *δ* was calculated analogously.

### Sound features other than pitch

To test whether substitution-induced changes of the targeted syllable were specific to pitch, we also inspected other sound features including syllable duration, amplitude modulation (AM), frequency modulation (FM), and entropy. Syllable duration was defined as the interval between consecutive threshold crossings of the root-mean-square (RMS) sound waveform, where the threshold for each animal was kept constant for all days analyzed. AM, FM, and entropy were computed as means over the entire syllable. We combined subs-low and subs-high birds by multiplying feature values in subs-low birds by −1 to account for the anti-symmetry between treatments. As a group, we compared the feature d′ values between the last LO day and the last day of the baseline (paired two-tailed *t*-test), Supplementary Fig. 1a.

### Pitch of non-targeted syllables

To test whether systematic pitch changes were restricted to the targeted syllable, we also inspected harmonic non-targeted syllables. In total, we found 10 such syllables in 5 birds. On these syllables as a group, we tested whether the pitch differences (d values) between the last day of substitution and the last day of baseline on average was different from zero. Again, we multiplied d′ values in subs-low birds by −1 to account for the anti-symmetry between treatments, Supplementary Fig. 1a. Pitch changes in non-targeted syllables across this time period were not different from zero: average d′ = 0.01, *P* = 0.43, tstat = 0.82, df = 9, two-tailed *t*-test, *N* = 10 syllables from 5 birds).

### Control (unsubs and noLO) birds

To evaluate whether an individual bird responded to substitution, we compared the daily pitch changes $\left\{ {d^{\prime}_{i - 1,i}} \right\}_{i \in {\mathrm{LO}}}$ of its targeted syllable to daily pitch changes in control birds (not exposed to LO). In subs birds, the control group was formed by 12 deaf (unsubs) birds, and in LO birds, the control group was formed by 12 hearing (noLO) birds. To account for possible pitch drifts caused by deafening or by time spent in the experimental chamber, the time window for pitch analysis in unsubs birds was matched to the substitution period in the subs bird, i.e., the first day analyzed in control birds occurred at the same time lag after deafening as the first LO day. Also, the number of days analyzed was identical in subs birds and unsubs birds (same for LO and noLO birds). To enforce robust statistics of pitch responses, we paired a subs bird only to unsubs controls that produced more than 100 song motifs on each day during the matched time periods. Two unsubs birds had to be excluded because they produced fewer than 100 renditions of the targeted syllable on days 11 and 12 after deafening (resulting in a total of 10 unsubs birds).

### Statistical testing of pitch responses

To test whether an individual bird significantly changed its pitch in response to LO, we compared all its daily pitch changes during the LO period to all daily pitch changes in control birds in matched time windows (at significance level *p* = 0.05, without correction for multiple comparisons, two-tailed two-sample *t*-test, indicated by asterisks in Figs. 1g, 2c).

For the population analysis, we compared daily pitch changes in all subs birds against all unsubs controls (same for LO and noLO birds). We randomly paired the 10 unsubs birds (dark gray bars in Fig. 1g) with the 10 subs birds (under the constraint that analysis days could be temporally matched). The pairing is depicted in Fig. 1g such that bird 11 was paired with subs bird 1, bird 12 with subs bird 2, etc. We did the same for the 12 LO birds in Fig. 2c, i.e., bird 13 was paired with LO bird 1, bird 14 was paired with LO bird 2, etc. All pairings were time-matched, i.e., the early (baseline, light gray bars in Fig. 1g) and late time periods in controls were defined according to the baseline and LO periods in the treated bird.

To verify that we did not observe a spurious effect because of a single choice of random pairing, we randomly paired the birds 1000 times, always ensuring that analysis days were temporally matched (not all pairings of birds were possible because of differences in experiment duration). We first matched the control birds with the least possible matching partners and so forth. In the case of singing rate (see below) there were more unsubs birds than subs birds. In this case, we first matched each subs bird to a random unsubs bird (without replacement) and then matched the two remaining unsubs birds randomly to two subs birds. In the result section, we report the *p*-values for one random pairing corresponding to the data shown in the figures.

### Linear mixed-effect model

To test whether subs/LO birds exhibited a common direction of pitch change (either towards the LO pitch zone or away from it), we modeled daily pitch changes $d^{\prime j}_{i - 1,i}$ in bird *j* during LO using a linear mixed-effect model:

$$d^{\prime j}_{i - 1,i} = b\vartheta _i + a\theta _i + d\varphi _i + r_j,$$

where the three fixed effect terms *b*, *a*, and *d* common to all birds were: the daily pitch change *b* during baseline ($\vartheta _i = 1$ if day *i* is during baseline and $\vartheta _i = 0$ otherwise), the pitch drift *a* without LO (*θ*_*i*_ = 1 in control birds if days *i* and *i*−1 occurred after baseline and *θ*_*i*_ = 0 otherwise), and the daily pitch change *d* caused by LO (*φ*_*i*_ = 1 for LO-high and *φ*_*i*_ = −1 for LO-low birds, provided both days *i*−1 and *i* were LO days). The *r*_*j*_ are zero-mean Gaussian noise terms that account for variability among birds. We separately fitted a linear mixed-effect model to deaf and to hearing birds.

We found the results displayed in Figs. 1h and 2d to be qualitatively unchanged when we either changed the model such that *a* and *d* describe changes relative to baseline ($\vartheta _i = 1$ for all days *i*) or when we reduced the model to two fixed effects (combining the terms *b* and *a* into a single term describing spontaneous pitch drift during baseline in subs/LO birds and on all days in control animals).

### Singing rate

To inspect the effects of light off on singing rate in hearing and in deaf birds, we measured the change in singing rate as the average number of targeted syllables at the end of the subs/LO period (average over last three days) subtracted by the average number on the last three days of baseline (Fig. 4a). We obtained qualitatively similar results when we used the normalized change in singing rate obtained by dividing the change in singing rate from each bird by the average syllable count on its last three days of baseline. For this analysis, we included all birds, including the two deaf control birds that stopped singing and could not be time-matched to any of the subs birds in the pitch-response analysis. For three birds, our recording system crashed and did not record vocalizations for one to three days. In these cases, daily song numbers from the day before were taken for the analysis. We compared relative singing rates between subs birds (*n* = 10) and unsubs birds (*n* = 12) and between LO birds (*n* = 12) and noLO birds (*n* = 12) using a two-tailed two-sample t-test (Fig. 4a). The *t*-test was significant for 97% of the random pairing of subs and unsubs birds demonstrating a stable *p*-value.

We also fitted a single linear mixed-effect model to deaf and to hearing birds. We modeled the relative singing rate $n_i^j$ on day *i* (for *i* being the last three days of subs/LO) of subs/LO bird *j* as follows:

$$n_i^j = c + a\alpha _j + b\beta _j + d\gamma _j + r_j,$$

where the four fixed effect terms *a*, *b*, *c*, and *d* common to all birds are: a general offset *c*, a change *a* in singing rate due to deafening (*a*_*j*_ = 1 if bird *j* was deaf and *a*_*j*_ = 0 otherwise), a change *b* due to LO (*β*_*j*_ = 1 if bird *j* was exposed to subs/LO and *β*_*j*_ = 0 otherwise), and a change *d* due to the interaction between deafening and LO (*γ*_*j*_ = 1 if bird *j* was a deaf subs bird and *γ*_*j*_ = 0 otherwise). The *r*_*j*_ are zero-mean Gaussian noise terms that account for variability among birds. We found a significant interaction *d* between deafening and LO (*p* = 0.005 for the random pairs shown in Fig. 4a, 99.9% of random pairings resulted in a significant interaction *d*) and a non-significant effect *b* of LO (*p* = 0.28 for the random pairs shown in Fig. 4a, 0% of random pairings resulted in a significant effect). Results were qualitatively unchanged when the model had separate fixed effects for light off in hearing and in deaf birds (*β*_*j*_ = 1 only if bird *j* was hearing and exposed to LO).

### Song degradation

To assess song degradation caused by deafening (Supplementary Fig. 1b–f), we inspected non-targeted syllables, comparing renditions at the beginning and the end of the experiment. Tschida and Mooney showed that both entropy and entropy variance significantly change after deafening^24^. Mean entropy is a measure of syllable noisiness and variance entropy of syllable complexity. To follow suit and inspect mean and variance entropy, we first semi-automatically clustered all (non-targeted) syllables using a nearest neighbor approach in the spectrogram domain. We only considered syllables that were sung more than 100 times on each day (22 syllables in hearing birds and 19 syllables in deaf birds). We calculated for each syllable type the magnitude mean-entropy change as the absolute difference in mean entropy between the last day before deafening and the first day after LO ended. For hearing birds, we chose the first day analyzed such that the duration of the analysis window matched the window in deaf birds. As a result, the intervals between the first and last day of the experiment did not significantly differ between birds in the hearing and deaf groups (*p* = 0.25, tstat = −1.16, df = 39, two-tailed two-sample *t*-test, mean hearing = 29 days, mean deaf = 27 days). Thus, differences between hearing and deaf birds in Supplementary Fig. 1b–f were not due to differences in time spent in the recording chamber.

In agreement with Tschida and Mooney, we found a larger magnitude variance-entropy change in deaf than in hearing birds (difference 0.25, *p* = 0.005, tstat = 2.96, df = 39, two-tailed two-sample *t*-test, Supplementary Fig. 1c). However, we found no difference in magnitude mean-entropy change (*p* = 0.61, Supplementary Fig. 1b). Note that Tschida and Mooney did not perform time-matched comparisons against a group of hearing birds as we did, but they compared entropy to baseline measurements taken before deafening, implying that mean entropy changes in their study could have been caused by birds’ gradual adaptation to the recording chamber, irrespective of the deafening procedure.

For non-targeted syllables, we calculated the pitch coefficient of variation CV_*i*_ on day *i* as ${\mathrm{CV}}_i = 100\frac{{{\upsigma }}_{\mathrm{i}}}{{\bar p_i}}$. As we had done for targeted syllables, we calculated the pitch within a fixed 16 ms window during a harmonic part of the syllable (provided the latter existed, i.e., a harmonic part was found in 10/19 syllables in deaf animals and in 9/22 syllables in hearing animals). The difference between the coefficients of variation on the last day of deafening and on the first day after LO was larger in deaf birds than in hearing birds, Supplementary Fig. 1d.

To compute spectral changes due to deafening, we performed a bias-variance decomposition. To calculate spectrograms, we first tapered the sound waveform using a Hamming window of 512 samples. The windowed signal was transformed into a linear-power sound spectrogram using the discrete fast Fourier transform computed over segments of 512 samples and nonoverlaps of 128 samples (corresponding to 4 ms). The log-power sound spectrogram was then obtained by taking the natural logarithm of the linear-power sound spectrogram after adding an offset of 0.1 (corresponding roughly to the 75th percentile). We computed the spectrograms of non-targeted syllables within a time window defined by the duration of the shortest syllable rendition. To achieve robustness to low-frequency noise present in the recordings, we ignored the lowest 10 frequency bins corresponding to a frequency cutoff at 625 Hz. The spectrogram bias of a particular syllable was defined as the Euclidean distance between the average spectrograms on two separate days: on the last day before deafening and the first day after the end of the LO period. The spectrogram variance was defined as the average pixel-wise variance on a given day. There was no significant difference between hearing and deaf birds in terms of either spectrogram bias or variance (bias: *p* = 0.45, tstat = −0.77, df = 39, variance: *p* = 0.32, tstat = 1.01, df = 39, two-tailed two-sample *t*-test, Supplementary Fig. 1e, f). Thus, the substitution period was too short to lead to a major spectral song degradation.

### Temporal resolution of pitch changes

Inspired by the analysis of Charlesworth et al.^40^ in hearing birds exposed to white noise, we next assessed the temporal dynamics of pitch changes in response to light off. We computed pitch traces over the entire syllable in a sliding window of 16 ms and plotted their temporal statistics at a time resolution of 1 ms, Fig. 3a, b and Supplementary Fig. 2. In each bird, to compare pitch traces from the last day of light off with traces from the last day before light off, we computed d’ values between the two distributions at 1-ms time scale relative to the window of LO delivery (Fig. 3e, f).

### Reporting summary

Further information on research design is available in the Nature Research Reporting Summary linked to this article.

## Supplementary information

Supplementary Information

Reporting Summary

## Data Availability

The pitch data that support the findings of this study together with the MATLAB scripts to reproduce the analysis and figures are available at the ETH Research Collection with the identifier [data 10.3929/ethz-b-000431869]^60^. The raw data underlying the pitch measurement is not deposited due to its size but is available from the authors upon reasonable request. A reporting summary for this Article is available as a Supplementary Information file. Source data are provided with this paper.

## Source data

Source Data

## Code availability

Our custom code to calculate pitch based on the Harmonic Product Spectrum^59^ algorithm can be accessed from our GitLab repository under the following link: https://gitlab.ethz.ch/songbird/pitch_hps. The code for data analysis and simulation of a simple agent using SARSA are available from the ETH Research Collection with identifier [10.3929/ethz-b-000431869]^60^.
