# Supplementary material for: Sensory substitution reveals a manipulation bias
Source: Nat Commun. 2020 Nov 23;11:5940. doi: 10.1038/s41467-020-19686-w (PMC7684286; doi:10.1038/s41467-020-19686-w)
Supplement: Supplementary file 1 — Supplementary Information [file 41467_2020_19686_MOESM1_ESM.pdf]

Supplementary Information:

Sensory substitution reveals a manipulation bias

Zai et al.

Supplementary Methods

Reinforcement Valence in Markov Decision (MD) Models

We describe a computational model of spontaneous behavior wherein a singing bird behaves like a simple probabilistic agent. We express the model in a reinforcement learning (RL) framework, according to which birds try to maximize the total reward obtained from their songs. This reward encompasses both extrinsic and intrinsic components, the latter of which we try to estimate.

1. Markov Modeling Framework

We assume that singing forms a Markov process in which at time t $(t = 1, \ldots, T)$, the motor action a^t triggers the sensory state s^t according to the unknown Markov observation matrix $\vartheta_j(k) = P(s^t = k | a^t = j)$ which denotes the probability of observing sensory state k given motor action j. One can think of this observation matrix as modeling the vocal organ including the motor noise associated with singing. In the following, k is the index of all distinct sensory (visual and auditory) states, and j is the index of all distinct motor states.

Through song practice, the bird obtains information about this observation matrix. We assume it does so by keeping track of how often it chose a given action and how often it observed a given state as a result. When at time t, the bird produces action j^* and observes state k^*, the bird's action-state counter $C_{j,k}^t$ is updated as follows:

$$C_{j,k}^t = \begin{cases} \tau \cdot C_{j,k}^{t-1} + 1 & \text{for } k = k^* \text{ and } j = j^* \\ C_{j,k}^{t-1} & \text{otherwise} \end{cases}$$

Here, τ models a forgetting rate to mimic birds' limited counting ability. We assume that forgetting is triggered by actions, not by time, to agree with one-shot aversive conditioning that can be long lasting[1]. Based on this action-state counter $C_{j,k}^t$, we want to formulate an intrinsic reward such that a greedy bird will choose actions in agreement with the behavior of both hearing and deaf birds in our experiments.

2. Song Model

For simplicity, we assume the bird sings a motif composed of a single syllable which in turn is composed of three notes $i = 1,2,3$. Because adult zebra finch syllables are very stereotyped, these notes are always produced in the same order. The part of motor variability that is voluntarily controlled by the

bird arises from each note having a distinct set of six actions associated with it. These actions span diverse note variants that are distinct in terms of their sound features including pitch. Thus, the bird's motor repertoire is composed in total of 18 different actions a_j $(j = 1, \dots, 18)$, where actions 1-6 are associated with note 1, actions $7 - 12$ with note 2, and actions $13 - 18$ with note 3.

To model motor noise that the bird cannot voluntarily control, we assume that each action probabilistically leads to one of three different sensory states, as depicted in Fig. 3B. The sensory states represent the actual sounds being produced. The center state has high probability (0.5) and the two flanking states have lower probabilities (0.25 each). The sensory states associated with a note tile the possible pitch values in a topographical manner such that a given state can be elicited by up to three different actions (because of motor noise). Without loss of generality, we assume that the sensory spaces of all three notes are distinct and nonoverlapping (complex syllable), which means we are dealing with a total of 25 different sensory states s_k $(k = 1, \dots, 25)$, where states $1 - 8$ are associated with note 1, states $9 - 16$ with note 2, states $17 - 24$ with note 3 and the 25th state corresponds to the silent state which hearing birds can never reach during singing.

In summary, when the bird sings note i, the Markov observation matrix $\vartheta_j(k)$ is given by

$$\vartheta_j(k) = \begin{cases} 0.5 & \text{for } k = k_0 \\ 0.25 & \text{for } k = k_0 - 1 \text{ or } k = k_0 + 1 \\ 0 & \text{otherwise,} \end{cases} \quad (1)$$

where $k_0 = j + 1 + 8(i - 1)$ is the index of the center state associated with the jth action. The roughly diagonal structure of the Markov observation matrix (Fig. 3B) amounts to assuming that the sensory and motor neural representations are well aligned with one another, which is the case for example when the brain forms a forward or inverse model of the vocal apparatus[2].

In note 2, half of the states (states $13 - 16$) trigger a brief LO event. The difference between hearing and deaf birds is that hearing birds experience all 24 sensory states (corresponding to different pitch values and light conditions) whereas deaf birds experience only two states (light off and neutral).

3. Intrinsic Reward from Impact

We write the total reward $R_{j^*}^t$ at time t and associated with action $a^t = j^*$ and observed state $s^t = k^*$ as the weighted sum of three terms: an exploration bonus $E_{j^*}^t$, a manipulation bonus $M_{j^*}^t$, and an extrinsic punishment r^t associated with the lighting condition:

$$R_{j^*}^t = E_{j^*}^t + M_{j^*}^t + r^t. \tag{2}$$

The three main terms are:

A. The exploration bonus $E_{j^*}^t$ is defined as the Markov information gain defined as the log Kullback-Leibler (KL) divergence $D_{\mathrm{KL}}\left(\hat{\vartheta}_{j^*}^{t-1} || \hat{\vartheta}_{j^*}^t\right)$ between $\hat{\vartheta}_j^t(k)$ and $\hat{\vartheta}_j^{t-1}(k)$[3,4]:

$$E_{j^*}^t = \log D_{\mathrm{KL}}\left(\hat{\vartheta}_{j^*}^{t-1} || \hat{\vartheta}_{j^*}^t\right), \text{ with } \quad D_{\mathrm{KL}}\left(\hat{\vartheta}_{j^*}^{t-1} || \hat{\vartheta}_{j^*}^t\right) = \sum_k \hat{\vartheta}_{j^*}^{t-1}(k) \log\left(\frac{\hat{\vartheta}_{j^*}^{t-1}(k)}{\hat{\vartheta}_{j^*}^t(k)}\right).$$

The agent's (biased) estimate $\hat{\vartheta}_j^t(k)$ of the Markov observation matrix $\vartheta_j^t(k)$ is given by the fraction of times a given state has been observed, $\hat{\vartheta}_j^t(k) = \frac{1+C_{j,k}^t}{1+\sum_l C_{j,l}^t}$. The exploration bonus is large when the bird explores rare actions, in which case the difference between the action-state counters $C_{j,k}^t$ and $C_{j,k}^{t-1}$ is large and so is the distance $D_{\mathrm{KL}}\left(\hat{\vartheta}_{j^*}^{t-1} || \hat{\vartheta}_{j^*}^t\right)$ between the estimated observation matrices $\hat{\vartheta}_{j^*}^{t-1}$ and $\hat{\vartheta}_{j^*}^t$.

B. The manipulation bonus $M_{j^*}^t$ associated with action j^* is defined as:

$$M_{j^*}^t = D_{\mathrm{KL}}\left(\hat{\vartheta}_0^t || \hat{\vartheta}_{j^*}^t\right),$$

where D_{KL} denotes the KL divergence $D_{\mathrm{KL}}\left(\hat{\vartheta}_0^t || \hat{\vartheta}_{j^*}^t\right) = \sum_k \hat{\vartheta}_0^t(\mathrm{k}) \log\left(\frac{\hat{\vartheta}_0^t(k)}{\hat{\vartheta}_{j^*}^t(k)}\right)$. In the context of our experiments, the light-off state can only be triggered by singing and thus $\hat{\vartheta}_0(k = \text{light off}) = 0$. When we set $\hat{\vartheta}_0^t(k = \text{light off}) = 0$ in the definition of the manipulation bonus, we obtain $M_{j^*}^t = -\log\left(\hat{\vartheta}_{j^*}^t(k = \text{light on})\right)$, i.e., the deaf bird will try to maximize the surprise of the light-on state by triggering LO as often as possible.

C. The external reward r^t is zero most of the time and has a fixed negative value $r < 0$ when the light goes off (because bird's vision is obstructed):

$$r^t = \begin{cases} r & \text{if note } i = 2 \text{ and } k^* \in \{13, \dots, 16\} \\ 0 & \text{otherwise.} \end{cases}$$

External rewards are maximized when the bird avoids actions associated with light off.

4. Action Choices in Reinforcement Learning Framework

We express the model in a simple SARSA framework [5] in which the agent makes greedy action choices

$$j^* = \underset{j \in \text{ note } i}{\operatorname{argmax}}\, Q_j^t, \tag{4}$$

by maximizing an action-value function Q_j^t that is defined as the running average reward[6]:

$$Q_{j^*}^t = Q_{j^*}^{t-1} + \alpha\left(R_{j^*}^t - Q_{j^*}^{t-1}\right), \tag{5}$$

with α being the learning rate. Initially, the action-value function $Q_j^t = 0$ is set to zero for all states j.

5. Intuition

In our stationary world, $\hat{\vartheta}_0^t(k = \text{light neutral}) = 1$ and the impact is maximized for actions j^* for which $\hat{\vartheta}_{j^*}^t(k = \text{light off}) = 1$, i.e., when the bird turns the light off. Maximizing the total expected reward $R_{j^*}^t$ leads to a less radical action choice, thanks to the exploration bonus. In practice, birds find some balance between impacting the world and probing diverse actions.

6. Model Details

To produce Fig. 3 and Fig. 4, we used the following simulation parameters: $T = 600$, $\tau = 0.99$, and $\alpha = 0.05$. In Fig. 3C, we plotted the fraction of syllables that triggered LO as a function of the negative extrinsic reinforcement r per LO. In Fig. 3D, we plotted the average action value $\langle Q_j^t \rangle_{T,j} = \frac{1}{18T} \sum_{j,t} Q_j^t$ for hearing and for deaf birds in the conditions with and without LO. We interpret high average action values as highly motivated birds that sing frequently and low action values as lowly motivated birds that sing infrequently.

For the simulation of dopaminergic neurons in Fig. 4, we defined the firing rate f to be proportional to reward prediction error[7]: $f \propto R_{j^*}^{t} - Q_{j^*}^{t-1}$.

Remarks

- Our definition of the manipulation bonus $M_{j^*}^{t}$ that we termed impact is inspired from[8], where impact is used as a norm for characterizing people's question-asking strategies.
- Except for motor noise inherent in the definition of $\vartheta_j(k)$, all aspects of the agent are deterministic.
- In simulations, $R_{j^*}^{t}$ should be non-positive, otherwise simulated birds get stuck on the one action for which the total reward is most positive. The logarithm in the definition of the exploration bonus tends to make sure that $R_{j^*}^{t}$ remains negative.
- None of the simulation results critically depends on the number of actions (i.e., six), the number of sensory states (i.e., eight) per note, and the amount of motor noise modeled. By contrast, it is important that the Markov observation matrix $\vartheta_j(k)$ in Equation (1) significantly differs between hearing and deaf birds (we assumed it is roughly diagonal and 24x18-dimensional in hearing birds in contrast to degenerate and 2x18-dimensional in deaf birds).
- The exploration bonus $E_{j^*}^{t}$ is not strictly needed in simulations. In a much simpler version of our model, the same qualitative results apply when $E_{j^*}^{t}$ is set to a fixed value, e.g. -6. The fixed subtraction of -6 on every chosen action in combination with Equation (4) guarantee that the bird will probe diverse actions, akin to maximizing the exploration bonus.

  Fig. 4 should be interpreted as a model of dopaminergic neuron firing reported in Gadagkar et al.[9]. In principle, the only necessary term in Equation (4) to model these results is r^t, which corresponds to the negative reward associated with white noise sound bursts (instead of LO). In this respect, the bonuses $E_{j^*}^{t}$ and $M_{j^*}^{t}$ mainly contribute variance.

  Note that we are not aware of any reports of visual responses in avian VTA neurons; however, visual responses in these neurons have been described[7] and therefore are likely to exist also in songbirds. Furthermore, although our model is expressed as a model of dopaminergic neurons, it is not meant to imply that all 3 terms in Equation (4) must necessarily be signaled by

dopaminergic neurons. It is conceivable that other neuromodulators such as serotonin have the function of signaling intrinsic rewards.

- We also tested the model's prediction for small positive reinforcement valence and found qualitative agreement of simulation results with data from the two light-on birds, Fig. S3, assuming that light-on is neutral or weakly appetitive (supported by hearing birds not showing an aversive response to strobe light[10]). Namely, for small positive valence of light-on, our model predicts that hearing birds barely respond to light on (contingency near 50%), whereas deaf birds show a significant preference by exhibiting light-on contingencies well above 50%, Fig. S3.

Supplementary Figures

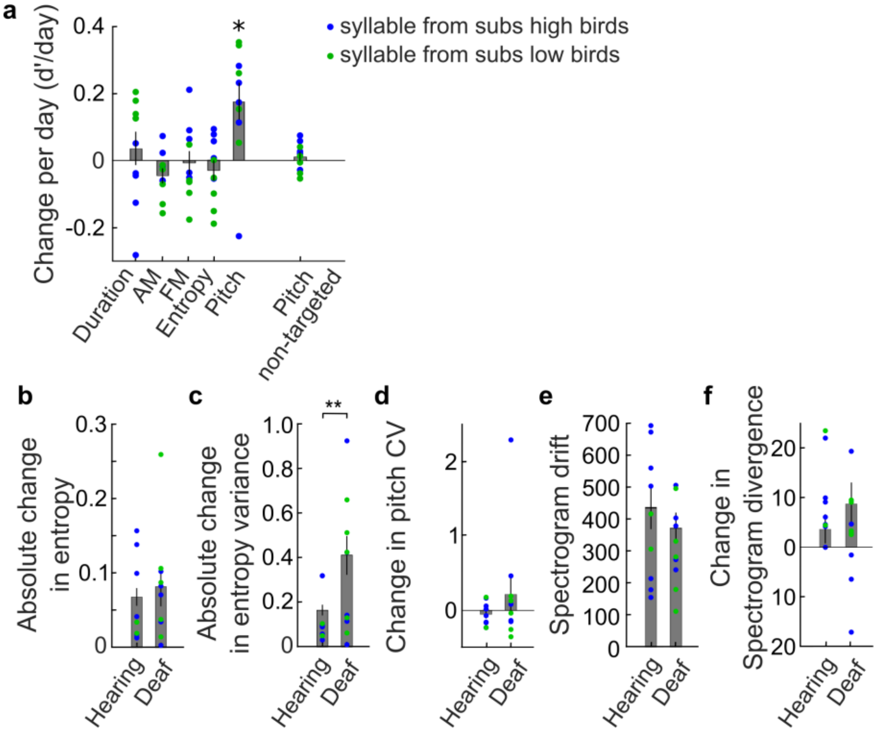

Supplementary Figure 1: Syllable dynamics in hearing and in deaf birds. a, In deaf birds (n=10 birds), pitch-contingent LO induced changes in pitch, but no other sound feature of the targeted syllable. Shown are changes (d' per day) across the time interval from the last day of baseline to the last day of LO exposure (interval average 11 days, range 8-17 days) for the following features: syllable duration, amplitude modulation (AM), frequency modulation (FM), entropy, and pitch. Also shown are pitch changes in non-targeted syllables (10 syllables from 5 birds). Bars indicate averages across birds and whiskers the standard errors of the mean. Before averaging, feature changes in subs-low birds (green dots) were multiplied by a factor -1 to align the changes with those of subs-high birds (blue dots). Significant nonzero changes of the target syllable were seen for pitch (p=0.01, two-tailed t-test, df=9, tstat=3.23, sd=0.17), but no other feature (p>0.05, two-tailed t-test). To compute d' per day in each animal, we divided the changes between the last day of baseline and last day of subs by the number of intermittent days. **b-f,** Non-targeted syllables diverged more in deaf birds (19 syllables from 7 birds in b, c, e, f; and 10 syllables from 5 birds in d) than in hearing birds (22 syllables from 8 birds in b, c, e, f; and 9 syllables from 6 birds in d). In deaf birds, the bars indicate the mean syllable changes across the time interval from the last day before deafening to the first day after LO exposure (interval average 27 days, range 23-33 days for b, c, e, and f; and average 26 days, range 23-33 days for d). In hearing birds, changes are measured across roughly equal time intervals. Shown are **b** entropy, **c** entropy variance, **d**

pitch coefficient of variation (CV), **e** drift in mean spectrogram, **f** spectrogram divergence. The error bars indicate the standard errors of the means and the stars indicate p value ('**': p=0. 005, tstat=2.96, df=39, 41 syllables from 15 birds, two sample two-tailed t-test).

Supplementary Note

Testing for a potential bias towards lower pitch in response to subs

We tested whether there could be a potential bias towards lower pitch in subs birds because 4/5 subs-low birds significantly changed their pitch downward and whereas only 2/5 subs-high birds changed their pitch significantly upward and one subs high-bird changes its pitch significantly downward (see Fig. 1g).

First, in targeted syllables in the 10 birds in Fi. 1g, the average pitch change per day during subs was not significantly different from zero (-0.09 d'/day, p=0.36, df=9, tstat = -0.959). Second, when we estimated pitch drift of the targeted syllable during subs using a linear mixed effects model, the drift was not significantly different from zero. Namely, we modeled the pitch change $d'^{j}_{i-1,i}$ from day $i-1$ to day i in subs bird j in response to subs as follows

$$d'^{j}_{i-1,i} = b\vartheta_i + a\theta_i + d\varphi_i + r_j,$$

where the three fixed effect terms b, a, and d common to all birds were: the daily pitch change b during baseline ($\vartheta_i = 1$ if day i is during baseline and $\vartheta_i = 0$ otherwise), the pitch drift a during subs ($\theta_i = 1$ if days i and $i-1$ occurred after baseline and $\theta_i = 0$ otherwise), and the daily pitch change d caused by LO ($\varphi_i = 1$ for sub-high and $\varphi_i = -1$ for subs-low birds, provided both days $i-1$ and i were LO days). The r_j were zero-mean Gaussian noise terms that account for variability among birds. We found the fixed effect a to be negative but not significantly different from zero (a=-0.09, SE=0.06, p=0.10, DF=138, tstat=-1.66), revealing a trend but no significant drift towards lower pitch. Third, one light-on bird significantly shifted pitch up, the other bird significantly shifted pitch down, revealing no asymmetry in this experimental group. And fourth, in non-targeted syllables (in 10 subs birds in Fig. 1g), we found a non-significant pitch increase (d'/day = 0.02$\pm$0.04, p=0.14, df=9, tstat=1.61, 10 non-targeted syllables from 5 birds, disregarding the LO contingency, Supplementary Figure 1a), though note the caveat that 5 non-targeted syllables were from subs-high birds and 5 non-targeted syllables were from subs-low birds, implying a pseudo-replication because of possible correlations among syllables within individual birds. Nevertheless, in combination, we found no support that light off induces a bias towards pitch decreases, rather this asymmetry can likely be ascribed to a small sample effect.

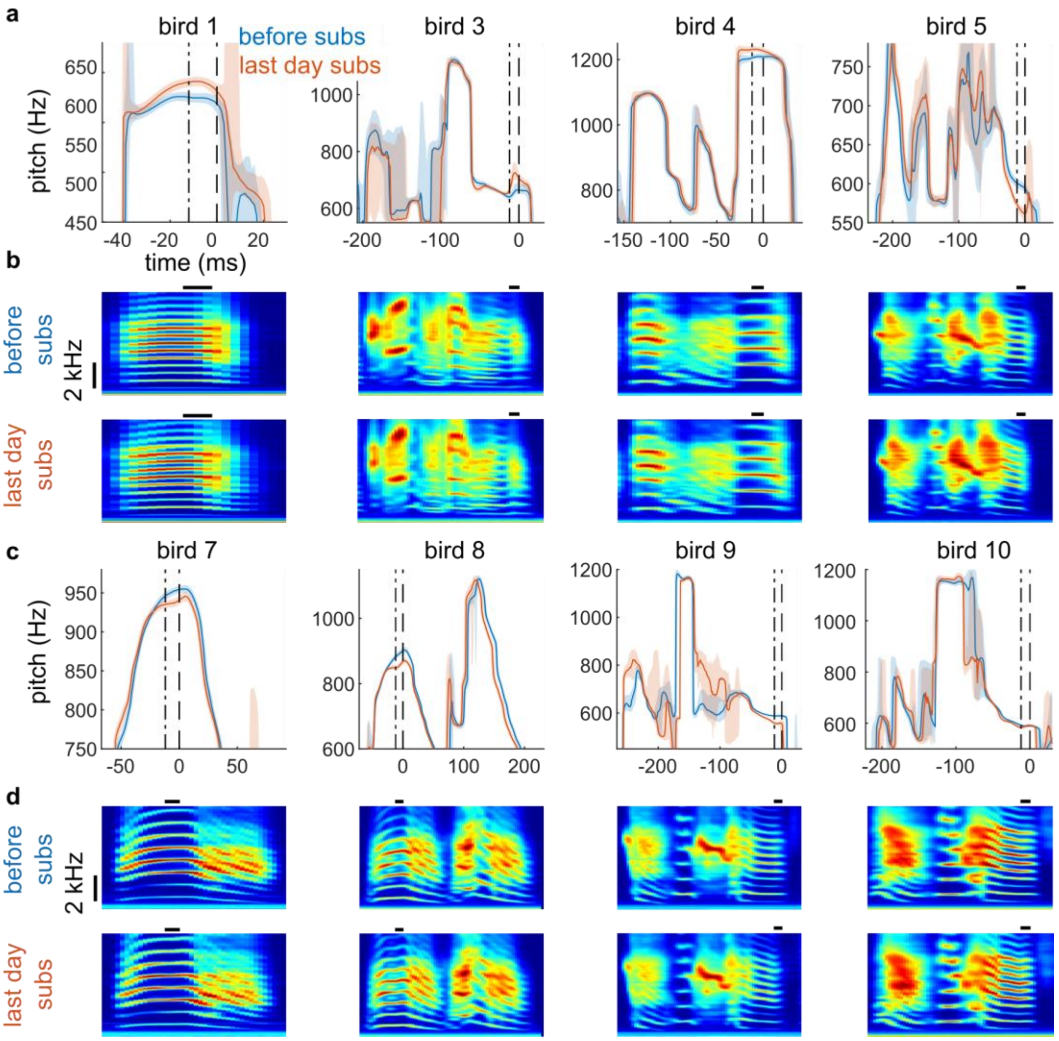

Supplementary Figure 2: Within-syllable pitch trajectories in the n=8 additional subs birds not shown in Fig 3. a and **c**, median (solid line) and quantiles (shaded area) of within-syllable pitch before substitution (before subs, blue) and on the last day of substitution (last day subs, red) in subs-high birds (a,b) and in sub-low birds (c,d). The two dashed vertical lines show the window within which the pitch was calculated to determine whether light was switched off. The birds are ordered according to the blue/green bars in Fig. 1g. **b** and **d**. Average spectrogram of target syllable before (top row) and after (bottom row) the light off paradigm. Same time axis as in panel a. Horizontal black line shows the window where pitch is calculated.

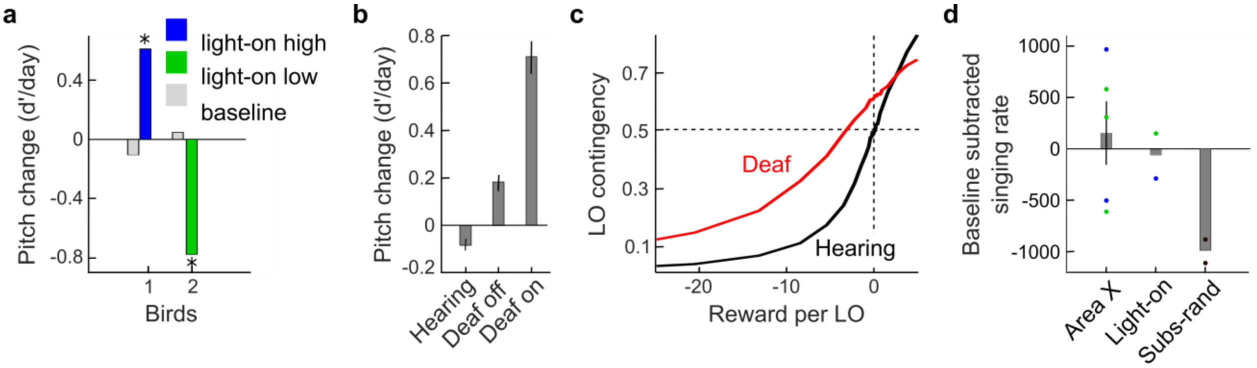

Supplementary Figure 3: Strong appetitive response to light-on stimuli in deaf birds. a, Histograms of average daily pitch changes during substitution in a bird with high-pitch light-on substitution (blue) and in a bird with low-pitch light-on substitution (green). The light grey bars to the left of the colored bars indicate the average daily pitch change for each syllable during the last 5 baseline days. The asterisks indicate significant pitch changes compared to controls (two sample, two-sided t-test, $p=8*10^{-7}$, df=48, tstat = -5.67 for bird 1 and $p=2*10^{-8}$, df=70, tstat = 6.37 for bird 2, see Methods) **b**, Same figure as Fig 2e with added light-on birds. Average directed pitch changes in LO (hearing, 144 days), subs light-off (Deaf off, 102 days), and subs light-on (Deaf on, 13 days) birds. The error bars indicate the standard errors of the mean. Deaf on birds changed their pitch towards the LO pitch zone. **c,** same as Fig. 4d but extended to positive reinforcement values r per LO. For small positive reinforcement values, hearing birds barely respond to LO (contingency around 50%, black line) whereas deaf birds reach LO contingencies high above 50% (red line). In this panel, for positive r, LO should stand for light on. **d,** Average changes in singing rate in deaf birds with Area X lesions and pitch-contingent LO feedback (n=5, Area X), in deaf birds with pitch-contingent light-on feedback (n=2, light-on), and in deaf birds with random LO feedback (n=2, subs-rand). Dots represent individual birds with subs-high birds shown in blue, subs-low birds in green, and subs -rand birds in black. Changes are reported relative to the average singing rate on the last three days of baseline. The Area-X data is taken from the last three days of light off, the light-on data from the last three days of light on, and the subs-rand data from days 9-11 of light off. Area X lesioned birds sung on average 154 more syllables than during baseline (p=0.09, tstat=-1.85, df=13, n=5 Area X lesioned subs and n=10 random unsubs birds, two unsubs birds could not be matched to any Area X lesioned birds, in 15% of random pairing the p-value was significant).

Supplementary References

1. Lee-Teng, E. & Sherman, S. M. Memory consolidation of one-trial learning in chicks. *Proc. Natl. Acad. Sci. USA* **56,** 926–931 (1966).

2. Hanuschkin, A., Ganguli, S. & Hahnloser, R. H. R. A Hebbian learning rule gives rise to mirror neurons and links them to control theoretic inverse models. *Front. Neural Circuits* **7,** 106 (2013).

3. Little, D. Y. & Sommer, F. T. Learning and exploration in action-perception loops. *Front. Neural Circuits* **7,** 37 (2013).

4. Sutton, R. S. in *Machine learning proceedings 1990* 216–224 (Elsevier, 1990). doi:10.1016/B978-1-55860-141-3.50030-4

5. Rummery, G. A. & Niranjan, M. *On-line Q-learning using connectionist systems*. (mi.eng.cam.ac.uk, 1994).

6. Watkins, C. J. C. H. & Dayan, P. Q-learning. *Mach Learn* **8,** 279–292 (1992).

7. Schultz, W., Dayan, P. & Montague, P. R. A neural substrate of prediction and reward. *Science* **275,** 1593–1599 (1997).

8. Nelson, J. D. Finding useful questions: on Bayesian diagnosticity, probability, impact, and information gain. *Psychol. Rev.* **112,** 979–999 (2005).

9. Gadagkar, V. *et al.* Dopamine neurons encode performance error in singing birds. *Science* **354,** 1278–1282 (2016).

10. Murdoch, D., Chen, R. & Goldberg, J. H. Place preference and vocal learning rely on distinct reinforcers in songbirds. *Sci. Rep.* **8,** 6766 (2018).
