# Supplementary material for: Sensory substitution reveals a manipulation bias
Source: Nat Commun. 2020 Nov 23;11:5940. doi: 10.1038/s41467-020-19686-w (PMC7684286; doi:10.1038/s41467-020-19686-w)
Supplement: Supplementary file 2 — Reporting Summary [file 41467_2020_19686_MOESM2_ESM.pdf]

nature research

Corresponding author(s): Richard H.R. Hahnloser

Last updated by author(s): Aug 18, 2020

Reporting Summary

Nature Research wishes to improve the reproducibility of the work that we publish. This form provides structure for consistency and transparency in reporting. For further information on Nature Research policies, see Authors & Referees and the Editorial Policy Checklist.

Statistics

For all statistical analyses, confirm that the following items are present in the figure legend, table legend, main text, or Methods section.

n/a	Confirmed	
☐	☒	The exact sample size (*n*) for each experimental group/condition, given as a discrete number and unit of measurement
☐	☒	A statement on whether measurements were taken from distinct samples or whether the same sample was measured repeatedly
☐	☒	The statistical test(s) used AND whether they are one- or two-sided<br>*Only common tests should be described solely by name; describe more complex techniques in the Methods section.*
☐	☒	A description of all covariates tested
☐	☒	A description of any assumptions or corrections, such as tests of normality and adjustment for multiple comparisons
☐	☒	A full description of the statistical parameters including central tendency (e.g. means) or other basic estimates (e.g. regression coefficient) AND variation (e.g. standard deviation) or associated estimates of uncertainty (e.g. confidence intervals)
☐	☒	For null hypothesis testing, the test statistic (e.g. *F*, *t*, *r*) with confidence intervals, effect sizes, degrees of freedom and *P* value noted<br>*Give P values as exact values whenever suitable.*
☒	☐	For Bayesian analysis, information on the choice of priors and Markov chain Monte Carlo settings
☒	☐	For hierarchical and complex designs, identification of the appropriate level for tests and full reporting of outcomes
☐	☒	Estimates of effect sizes (e.g. Cohen's *d*, Pearson's *r*), indicating how they were calculated

Our web collection on statistics for biologists contains articles on many of the points above.

Software and code

Policy information about availability of computer code

Data collection	We used custom code from our lab written in LabVIEW (National Instruments, Inc.) for data collection.
Data analysis	For data analysis we used custom MATLAB R2019b code from our lab and to extract sound features we used a modified version of a MATLAB library of Sound Analysis Pro (http://soundanalysispro.com/matlab-library). Our custom code to calculate pitch based on the Harmonic Product Spectrum59 algorithm can be accessed from our GitLab repository under the following link: https://gitlab.ethz.ch/songbird/pitch_hps. The MATLAB scripts used for analysis and the simulations using SARSA are available at the ETH Research Collection: DOI: 10.3929/ethz-b-000431869

For manuscripts utilizing custom algorithms or software that are central to the research but not yet described in published literature, software must be made available to editors/reviewers. We strongly encourage code deposition in a community repository (e.g. GitHub). See the Nature Research guidelines for submitting code & software for further information.

Data

Policy information about availability of data

All manuscripts must include a data availability statement. This statement should provide the following information, where applicable:

- Accession codes, unique identifiers, or web links for publicly available datasets
- A list of figures that have associated raw data
- A description of any restrictions on data availability

The dataset acquired for this article (Fig.1, Fig.2 Fig.3, Fig.4 and supplementary Fig. 1, 2 and 3) will be published at the ETH Research Collection (DOI: 10.3929/ethz-b-000431869) upon acceptance.

Field-specific reporting

Please select the one below that is the best fit for your research. If you are not sure, read the appropriate sections before making your selection.

☒ Life sciences ☐ Behavioural & social sciences ☐ Ecological, evolutionary & environmental sciences

For a reference copy of the document with all sections, see nature.com/documents/nr-reporting-summary-flat.pdf

Life sciences study design

All studies must disclose on these points even when the disclosure is negative.

Sample size	Sample sizes were not predetermined since the experiments were exploratory in nature. Animal numbers were chosen according to the standard in the field and in accordance with the animal experimentation license. We did not perform any itermin evaluation of the results.
Data exclusions	No data was excluded except from two deaf control birds because they produced fewer than 100 renditions 11 and 12 days after deafening and could therefore not be time matched to any experimental birds to measure changes in pitch. These two birds were included for the analysis on singing rate.
Replication	The behavioral experiment was repeated independently over many individuals all of which are reported in the study. No individual was used twice except for one bird. This bird first shifted its pitch away from LO while hearing (Figure 2) and then after deafening shifted its pitch towards LO (Figure 1).
Randomization	Birds are chosen by age, sex and accessability in our breeding facilities. Their group identity was determined before the beginning of the experiment without any knowledge about their song or learning ability.
Blinding	The investigators were not blinded to group allocation during data collection since deaf birds behave differently to hearing birds and thus blinding is not possible. In any case, blinding was unlikely to make a difference because our behavioral paradigm runs as a fully automated system with minimal involvement of the experimenter. During data analysis, the manual work includes visual removal of misdetections. Investigators were not blinded in respect to the identity of the animal during this process. However, we do not expect a bias there because the relevant experimental quantity (pitch) is irrelevant and unknown during this process and thus unlikely to be influenced by removal of misdetections. Histological confirmation of the lesion extent and location was performed by an investigator that was blinded in respect to the animal identity.

Reporting for specific materials, systems and methods

We require information from authors about some types of materials, experimental systems and methods used in many studies. Here, indicate whether each material, system or method listed is relevant to your study. If you are not sure if a list item applies to your research, read the appropriate section before selecting a response.

Materials & experimental systems

n/a	Involved in the study
☒	☐ Antibodies
☒	☐ Eukaryotic cell lines
☒	☐ Palaeontology
☐	☒ Animals and other organisms
☒	☐ Human research participants
☒	☐ Clinical data

Methods

n/a	Involved in the study
☒	☐ ChIP-seq
☒	☐ Flow cytometry
☒	☐ MRI-based neuroimaging

Animals and other organisms

Policy information about studies involving animals; ARRIVE guidelines recommended for reporting animal research

Laboratory animals	We used adult male zebra finches (Taeniopygia guttata) raised in our breeding facilities in Zurich (Switzerland) and Orsay (France). At the beginning of the experiment, birds were between 90 and 200 days old.
Wild animals	No wild animals were used in this study.
Field-collected samples	No field collected samples were used in this study.
Ethics oversight	All experimental procedures were approved by the Veterinary Office of the Canton of Zurich or by the French Ministry of Research and the ethical committee Paris-Sud and Centre (CEEA N°59, project 2017-12).

Note that full information on the approval of the study protocol must also be provided in the manuscript.
